# Divergent water use efficiency trends among eastern North American temperate tree species

**DOI:** 10.1007/s00442-025-05753-w

**Published:** 2025-07-29

**Authors:** Jacob D. Malcomb, Howard E. Epstein, Matthew A. Vadeboncoeur, Daniel L. Druckenbrod, Matthew Lanning, Lixin Wang, Heidi Asbjornsen, Todd M. Scanlon

**Affiliations:** 1 Department of Environmental Sciences, University of Virginia, Charlottesville, VA USA; 2 Earth Systems Research Center, University of New Hampshire, Durham, NH USA; 3 Department of Earth and Chemical Sciences, Rider University, Lawrenceville, NJ USA; 4 Department of Geology and Environmental Sciences, State University of New York at Fredonia, Fredonia, NY USA; 5 Department of Earth Sciences, Indiana University Indianapolis, Indianapolis, IN USA; 6 Department of Natural Resources and the Environment, University of New Hampshire, Durham, NH USA

**Keywords:** Water use efficiency, Temperate forests, Nitrogen deposition, Sulfur deposition, Ozone, Plant functional types, Dendrochronology

## Abstract

Both theory and observations suggest that tree intrinsic water use efficiency (iWUE)—the ratio of photosynthetic carbon assimilation to stomatal conductance to water—increases with atmospheric CO_2_. However, the strength of this relationship varies across sites and species, prompting questions about additional physiological constraints and environmental controls on iWUE. In this study, we analyzed tree core carbon isotope ratios to examine trends in, and drivers of, iWUE in 12 tree species common to the temperate forests of eastern North America, where forests have experienced changes in CO_2_, climate, and atmospheric pollution in recent decades. Across all site-species combinations, we found that tree iWUE increased 22.3% between 1950 and 2011, coinciding with a 25.2% increase in atmospheric CO_2_. iWUE trajectories varied markedly among tree functional groups and within species across sites. Needleleaf evergreen iWUE increased until circa 2002 before declining in recent years, while iWUE of broadleaf deciduous species continued to increase. The analysis of environmental controls on iWUE trends revealed smaller increases in iWUE in trees subjected to higher atmospheric pollution loads. Our results suggest that tree functional characteristics and atmospheric pollution history influence tree response to atmospheric CO_2_, with implications for forest carbon and water balance in temperate regions.

**Supplementary Information:**

The online version contains supplementary material available at 10.1007/s00442-025-05753-w.

## Introduction

In terrestrial plants, photosynthetic carbon uptake is inextricably linked to loss of water via transpiration. The tradeoff between carbon gained and water lost is commonly expressed as intrinsic water use efficiency (iWUE), the ratio of photosynthetic carbon assimilation (*A*) to stomatal conductance to water (*g*_*s*_). Increases in iWUE as atmospheric CO_2_ (*C*_*a*_) concentrations rise are predicted by theory (e.g., Medlyn et al. 2011), and have been observed in CO_2_ enrichment experiments (e.g., Ainsworth and Rodgers 2007), eddy-covariance studies (e.g., Mastrotheodoros et al. 2017), and in studies that utilize tree ring carbon isotope ratios to derive iWUE over decadal to centennial time scales (Saurer et al. 2004), Frank et al. 2015; van der Sleen et al. 2015; Guerrieri et al. 2019). However, other recent evidence suggests that the effects of *C*_*a*_ on iWUE have weakened in recent decades (Belmecheri and Lavergne 2020; Adams et al. 2021), changes in iWUE have wide-ranging consequences for vegetation-climate feedbacks from ecosystem to global scales, including tree sensitivity to climate stressors (Heilman et al. 2021), atmospheric water vapor concentrations (Swann et al. 2016; Richardson et al. 2018), and water yields from forested catchments (Betts et al. 2007; KooperMan et al. 2018). Thus, accurately predicting how environmental changes affect tree stomatal behavior is essential to projecting future changes in terrestrial carbon, water, and energy cycles.

While there is strong evidence for CO_2_-driven enhancement of iWUE in terrestrial plants (Walker et al. 2021), increases in *C*_*a*_ have occurred alongside changes in other environmental variables, including climate (Rayback et al. 2020; Mathias and Thomas 2021) and atmospheric pollution (Savard et al. 2010; ThoMas et al. 2013; Holmes 2014), that can modulate tree response to changes in *C*_*a*_. Wet conditions tend to dampen iWUE response to elevated *C*_*a*_ (Levesque et al. 2017; Belmecheri et al. 2021), while iWUE tends to increase in trees experiencing increasing drought (Zhang et al. 2019; Kannenberg et al. 2021). These effects of climate on iWUE are consistent with stomatal optimization theory, which suggests that plants optimize stomatal regulation to maintain leaf water potential above a species-specific threshold (Cowan et al. 1982). When trees are not stressed by water supply or atmospheric demand, there is little advantage to using water efficiently and stomatal conductance is regulated to maximize photosynthesis.

Effects of air pollution—including ozone and the deposition of nitrogen (N) and sulfur (S)—on iWUE remain less clear, as these pollutants both directly impact leaf physiology and indirectly impact trees via deposition-driven changes in soil nutrient availability. Either mechanism may independently alter *A* and *g*_*s*_. Leaf exposure to N and S deposition may enhance iWUE by inducing stomatal closure, resulting in proportionally larger reductions in *g*_*s*_ than *A* (Bukata and Kyser 2007; ThoMas et al. 2013; Savard et al. 2020). In N-limited temperate forest ecosystems, N deposition may increase iWUE by enhancing *A* without a proportional or greater increase in *g*_*s*_ (Brooks and Mitchell 2011; Jennings et al. 2016; Gahrun et al. 2021; Mathias et al. 2023). On the other hand, chronic N and S deposition can drive soil acidification and base cation leaching (Driscoll et al. (Driscoll et al., 2001)), leading to plant nutrient deficiencies that impair *A* (St. Clair and Lynch 2005). The deposition-driven soil nutrient imbalances may result in declining iWUE when phosphorous (P) and/or base cations limit *A* (Huang et al. 2016), or when plants upregulate *g*_*s*_ to maintain sufficient mass flow of nutrients from the soil solution (Cramer et al. 2009; Lu et al. 2018; Lanning et al. 2019). While effects of N and S deposition on iWUE may be context-dependent, ozone is negatively associated with iWUE. Ozone causes oxidative stress that reduces *A* (Wittig et al. 2009) and may also impair stomatal function, leading to increases in *g*_*s*_ (McLaughlin et al. 2007). In the United States, declines in atmospheric ozone have been linked to increases in forest iWUE in recent decades (Holmes 2014).

In addition to climate and atmospheric pollution, tree iWUE response to increasing *C*_*a*_ also differs among species with different leaf morphologies and hydraulic traits. iWUE of conifer species tends to be higher than that of broadleaf deciduous species (Frank et al. 2015; Guerrieri et al. (Guerrieri et al. 2019)), and more sensitive to increasing *C*_*a*_ (Soh et al. 2019). These effects may be explained by lower mesophyll conductance in conifers, which makes them more sensitive to changes in *C*_*a*_ (Niinemets et al. 2011), or by the isohydric stomatal behavior typical of many conifer species, which causes them to close their stomata and maintain low rates of *g*_*s*_ as drought conditions develop (Brodribb et al. 2014). Among broadleaf deciduous species, iWUE is more sensitive to environmental changes in species that exhibit isohydric stomatal behavior compared to more anisohydric species, which regulate *g*_*s*_ more loosely under drought stress at greater risk of hydraulic failure (Yi et al. 2019). Xylem anatomy, which is closely related to stomatal behavior (Klein 2014), also plays a key role in regulating transpiration. Understanding how these tree structural and functional characteristics interact with environmental variables to influence iWUE is key to predicting how forests will respond to environmental change.

In this study, we examined trends in, and drivers of, tree iWUE in eight broadleaf deciduous species and four needleleaf evergreen species in the temperate mixed deciduous forests of the eastern Unites States, where trees have experienced concurrent changes in *C*_*a*_, climate, and atmospheric pollution in recent decades. While previous studies have examined how climate and atmospheric pollution influence temperate forest iWUE response to *C*_*a*_ (Levesque et al. 2017; Mathias and Thomas 2018; Maxwell et al. 2019; Rayback et al. 2020; Gharun et al. 2021; Mathias et al. 2023), this study includes a broader diversity of species than other studies to date and leverages a large tree ring network spanning environmental gradients to examine the trajectory of iWUE over time, and drivers of variation in iWUE trends across space. Analyzing 38 unique site-species chronologies, we compared iWUE trends across species with diverse hydraulic characteristics to address the following questions: (1) How have tree hydraulic traits influenced iWUE trajectories in northeastern North American temperate species since the mid-twentieth century? and (2) How have climate and atmospheric pollution influenced the magnitude of iWUE trends across sites? These analyses offer unique insights into regional-scale controls on temperate forest iWUE trends, and may improve our ability to model forest carbon and water balance in response to future anthropogenic change.

## Materials and methods

### Sampling sites

**Fig. 1:**
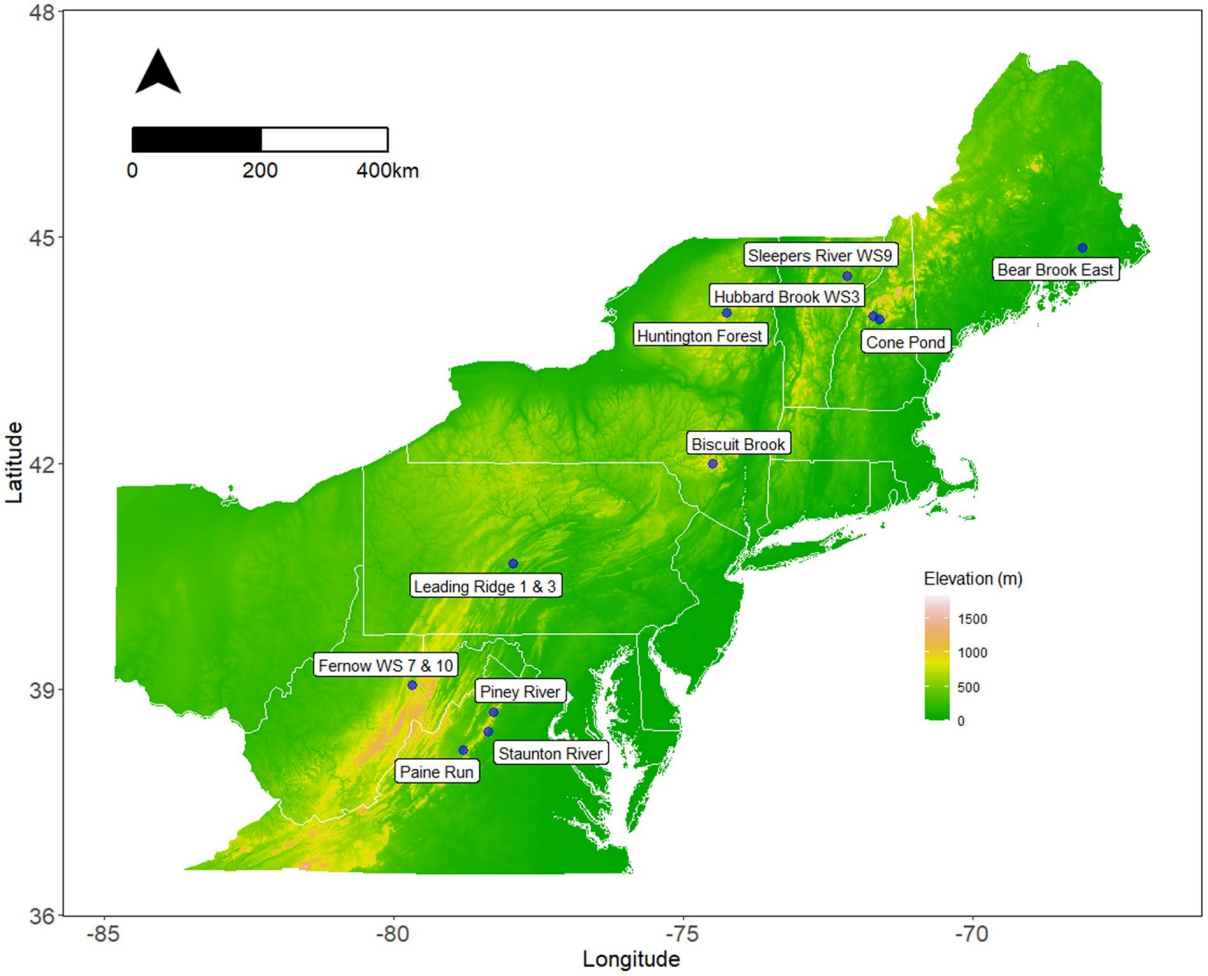
Tree sampling sites in the eastern United States. Tree cores used to derive tree ring δ^13^C chronologies were collected from 1 to 4 species at each site for a total of 38 unique site-species combinations. Details about species sampled at each site, and the number of trees in each iWUE chronology, can be found in Tables 1 and S1

The tree cores were collected from 1 to 4 species at each of 12 forested watershed sites in the eastern United States (Fig. 1, Table 1). The sites were selected based on the availability of long-term hydrologic records and ancillary forest composition, climate, and biogeochemical data. Some of these locations also host long-term manipulative experiments, but our analysis includes only unmanipulated control stands. The sites also span gradients of climate and atmospheric deposition. Mean June–August temperature ranged from 16.5 °C at Biscuit Brook, the coolest site, to 22.5 °C at Paine Run, the warmest site. Mean June–August precipitation ranged from 271 mm at Bear Brook, the driest site, to 428 mm at Fernow Experimental Forest, the wettest site. Watersheds at Fernow received the highest rates of atmospheric deposition (28.1 kg N + S ha^−1^ year^−1^), while Bear Brook received the lowest levels of annual atmospheric deposition (9.5 kg N + S ha^−1^ year^−1^) between 2000 and 2015 (Table 1). Mean June–August ozone concentrations were highest (0.059 ppm) at the three Shenandoah National Park Sites (Paine Run, Piney River, and Staunton River) and lowest at Cone Pond and Hubbard Brook (0.035 ppm).

**Table 1:**
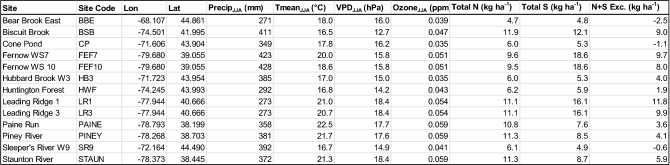
Mean climate and atmospheric pollution values for each site where trees were sampled

Precip_JJA_, Tmean_JJA_, and VPD_JJA_ represent the temporal mean of June–August precipitation, temperature, and maximum VPD calculated from 1980 to 2014. Ozone_JJA_ represents mean June–August ozone concentrations from 1993 to 2011, the common measurement period among all sites. Total *N* and total *S* are the mean of total annual (wet + dry) deposition from 2000 to 2015, the time period over which total deposition data is available nationwide. N + S Exc. represents the mean annual exceedance of N + S critical loads for soil acidification from 2000 to 2015

### Climate, deposition, and ozone data

The precipitation and temperature data were acquired from on-site meteorological stations at the Fernow Experimental Forest and Hubbard Brook Experimental Forest. For all other sites, precipitation and temperature data were acquired from PRISM (PRISM Climate Group, 2014). Daily maximum vapor pressure deficit (VPD_max_) data were also acquired from PRISM for all sites. Meteorological summer (June–August) total precipitation, mean temperature, and VPD_max_ were calculated annually for each site from 1980 to 2014. N and S deposition data were acquired from the National Atmospheric Deposition Program’s Total Deposition (Tdep) product (Schwede and Lear 2014), which combines wet deposition data from the National Trends Network (NTN) and dry deposition data from the Clean Air Status and Trends Network (CASTNET) to generate an estimate of total (wet + dry) deposition for the continental United States. Tdep raster data were clipped to watershed boundaries and annual watershed-scale estimates were calculated from 2000 to 2015. The deposition data were processed on an annual basis, since deposition may have direct effects on foliar health and stomatal function (Sayre and Fahey 1999), and indirect, cumulative effects on soil nutrients (Driscoll et al. (Driscoll et al., 2001)). Thus, deposition received outside of the growing season can still influence vegetation physiology. Exceedance of N and S deposition critical loads for forest soil acidification was calculated as the difference between total N and S deposition (2000–2015) and critical load values for each site (McNulty et al. 2007). Since critical load exceedance primarily reflects impacts of deposition on soils, this variable was selected to assess whether deposition effects were primarily related to direct or indirect impacts of deposition. Ozone data were acquired from the EPA Air Quality System (https://www.epa.gov/aqs) from the nearest monitoring station to each site that had at least 10 years of ozone data (Table S1). Peak growing season (June–August) ozone concentrations were calculated annually for each site between 1993 and 2011, the common period across all ozone monitoring sites.

### Tree ring sampling and isotopic analyses

This study incorporates data from tree cores collected specifically for this study, and leverages iWUE chronologies originally developed for other studies within the regional tree ring network. In each watershed, two tree cores were collected from 15 to 20 individual trees of each focal species. Further information about each chronology can be found in Table S2. The tree cores were air-dried, hand-sanded, cross-dated, and measured using standard dendrochronological procedures (Speer 2012), but not mounted with glue to facilitate subsampling of cores for isotope analysis. Cross dating was statistically validated using COFECHA (Holmes 1983).

A subset of 5–8 trees for each site-species combination were selected for carbon isotope analysis based on our degree of confidence in the correct dating as indicated by the absence of credible cross-dating flags in COFECHA and the correlation of chronologies with the master series. The isotope cores were sliced (or split) manually and shredded using razor blades. To maintain a manageable number of isotope samples (but still enough to assess temporal patterns), the isotope chronologies were processed in different ways, depending on the site, facility where samples were analyzed, and other study objectives for the samples. Samples from FEF10, LR1 and LR3, BSB, HWF, SR9, CP, and HB3 were combined by species and processed together as annual composite samples beginning in 1950 (or 1960 in the case of SR9 due to the age of the trees). Samples from PAINE, PINEY, and STAUN were processed as individual cores in 5-year increments beginning in 1951, while samples from FEF7 and BBE were processed as individual cores in 5-year increments beginning in 1979 and 1981, respectively. Individual rings or multiple-ring core segments were cut from each selected core using a razor blade under a microscope. The samples were comprised of whole rings for diffuse-porous hardwood species (ACRU, ACSA, FAGR, LITU) and latewood only for conifers (PIRI, PIRU, PIST, TSCA) and ring-porous hardwoods (FRAM, QURU, QUMO), as latewood isotopic composition should primarily reflect the environmental signal from that growth year (Jennings et al. 2016). Even though QUMO is ring-porous, the small ring widths (e.g., often < 200 µm) that were common for this species prevented the separation of early and latewood. For the site-species combinations where multiple δ^13^C chronologies were developed per watershed (BBE, FEF7, PAINE, PINEY, STAUN), a δ^13^C mean chronology was calculated and used in subsequent analyses.

The samples from CP and BBE were analyzed for δ^13^C on an Isoprime IRMS (Elementar, Langenselbold, Germany) at the University of New Hampshire (UNH) Instrumentation Center. Samples from BSB, FEF10, HBEF, HWF, LR1, LR3, and SR9 were run on a Thermo-Finnegan Delta XP IRMS at Washington State University (Pullman, WA). All samples analyzed at UNH and Washington State University were extracted to α-cellulose according to methods described by Leavitt and Danzer (1993). To ensure analytical consistency, reference wood and cellulose were analyzed both the UNH and Washington State University instruments. The reference samples ran similarly to within 0.1 ‰. Precision of duplicate sample analyses was also generally better than 0.1‰.

For samples from FEF7, PAINE, PINEY, and STAUN, α-cellulose was extracted using the Brendel method (Brendel et al. 2000) and analyzed for δ^13^C at Indiana University-Purdue University Indianapolis (IUPUI) on a Costech elemental analyzer coupled with Delta V IRMS. Precision of standards and replicate samples IUPUI was ≤ 0.2‰. Reference cellulose (Sigma-Aldrich) was included in each analytic batch to ensure data consistency between different lab facilities. After comparing δ^13^C values for reference wood from which cellulose was extracted and analyzed at each lab, IUPUI δ^13^C samples were adjusted by -0.9 ‰ to correct for bias caused by the different extraction methods between the two labs. Isotope data and more detailed field and lab methods are available from the Environmental Data Initiative (Scanlon et al. 2022).

### Calculation of tree iWUE

The stable *C* isotope composition of tree ring cellulose (*δ*^13^*C*_*t*_; ‰) was calculated using:

1 $${\delta }^{13}{C}_{t} = ({R}_{\text{sample}} / {R}_{\text{standard}} -1)\times 1-1000,$$

where *R*_sample_ and *R*_standard_ represent the ^13^C:^12^C ratios in the plant tissue and the PeeDee belemnite standard, respectively. *δ*^13^*C*_*t*_ was used to calculate carbon isotope discrimination (Δ^13^*C*) using

2 $${\Delta }^{13 }C=\frac{\left({\delta }^{13}{C}_{a} - \left({\delta }^{13}{C}_{t} - d\right)\right)}{\left(1+ \frac{\left({\delta }^{13}{C}_{t} - d\right)}{1000}\right)},$$

where *δ*^13^*C*_atm_ is the carbon isotopic signature of atmospheric CO_2_ and *d* is the post-photosynthetic fractionation correction factor (2.1 ± 1.2 ‰; Gessler et al. 2014; Lavergne et al. 2019). We note that the value of *d* varies across species and can be as high as 6‰ in the species included in this study (Lavergne et al. 2022). However, use of a constant value is warranted in this case since the iWUE analyses are focused on temporal trends, which would be unaffected by the application of a constant offset. We used *C*_*a*_ and ${\delta }^{13}{C}_{a}$ time series from Belmecheri and Lavergne (2020).

iWUE was calculated using the R package *isocalcR* using the mesophyll formulation using the default parameters (Mathias and Hurdiburg 2022). This method accounts for fractionation during mesophyll conductance and is less likely to result in overestimation of iWUE (Ma et al. 2021; Gong et al. 2022). The mesophyll conductance method requires elevation and temperature data—watershed mean elevation and annual June–August mean temperature were used for these parameters. The mesophyll formulation estimates CO_2_ concentrations at the chloroplast (C_*c*_), which may be substituted for the value of leaf intercellular CO_2_ concentrations (*C*_*i*_) used in the iWUE calculations developed by Farqhuar et al. (1989). Tree iWUE is defined as the ratio of photosynthetic carbon assimilation (*A*) to stomatal conductance to water vapor (*g*_*s*_):

3 $${\text{iWUE}= \frac{A}{g}}_{s}= \frac{{C}_{a}-{C}_{c}}{1.6},$$

where *C*_*a*_ and *C*_*c*_ are the atmospheric and chloroplast CO_2_ concentrations, respectively, and 1.6 is the diffusivity of water vapor relative to CO_2_.

### Data analyses

To maintain a standard temporal resolution, all annual iWUE chronologies were aggregated into 5-year segments prior to conducting statistical analyses (Figure S1). Both means and trends of tree iWUE were compared among species of different leaf functional types (broadleaf deciduous or needleleaf evergreen), xylem anatomies (diffuse porous, ring porous, or tracheid; Elliott et al. 2017), and general tendencies of stomatal behavior (isohydric or anisohydric; RoMan et al. 2015). Both linear and generalized additive models (GAMs) were used to assess trends in iWUE, using standardized chronologies for each site-species combination. Model fit was assessed using R^2^ and AIC. GAMs were fit using the “mgcv” R package (Wood 2011) with a cubic regression spline. Where visualization of GAMs suggested potential breakpoints in iWUE chronologies, piecewise regression using the R package *segmented* (Muggeo 2008) was used to detect statistical breakpoints in iWUE chronologies. Slopes of iWUE trends were compared among tree functional characteristics both before and after an identified breakpoint using the *emmeans* R package (Lenth 2017).

Because there was more substantial variability in iWUE trends among broadleaf deciduous species and sites compared to evergreen needleleaf species, subsequent analyses of environmental controls on iWUE trends examined only broadleaf deciduous species. We calculated Thiel-Sen slopes to estimate the temporal trend for each site-species combination between 1980 and 2014, the common period across all chronologies (the number of years in each chronology is listed in Table S2). This period was selected for several reasons: (1) to assess the influence of more recent environmental changes on iWUE; (2) to maximize the number of isotope chronologies eligible for analysis, since chronologies at BBE and FEF7 began in ~ 1980; and (3) to minimize tree age, size, and/or canopy position effects on carbon isotope discrimination (Leavitt 2010; Brienen et al. 2017; Vadeboncoeur et al. 2020) that are generally greatest in the early decades of tree development. Pearson’s correlation analyses were conducted to examine pairwise relationships between Thiel-Sen’s slopes for each site-species iWUE chronology and the study period means of environmental drivers at each site, and also correlations between the environmental drivers themselves.

The results of correlation analyses were used to inform variable selection in linear mixed effects models to identify environmental controls on the magnitude of iWUE trends across sites. Linear mixed effects models were fit using the R package *nlme* (Pinheiro et al. 2017), with iWUE slope (1980–2014) as the dependent variable and species as a random effect. Three models were assessed: a model including climate (mean growing season precipitation and VPD_max_) and air pollution variables (total N + S deposition and ozone), a model with only climate variables, and a model with only air pollution variables. Predictor variables were standardized prior to analyses to enable direct comparison of effect sizes. Multicollinearity among predictors was assessed by calculating the variance inflation factor (VIF) using the vif() function in the *car* package (Fox and Weisberg 2019). Fit and parsimony of models were assessed using corrected Akaike Information Criterion (AICc) and marginal and conditional R^2^ values, both calculated using the R package *MuMIn* (Bartoń 2017).

## Results

### iWUE values and temporal trends by tree functional traits

**Fig. 2:**
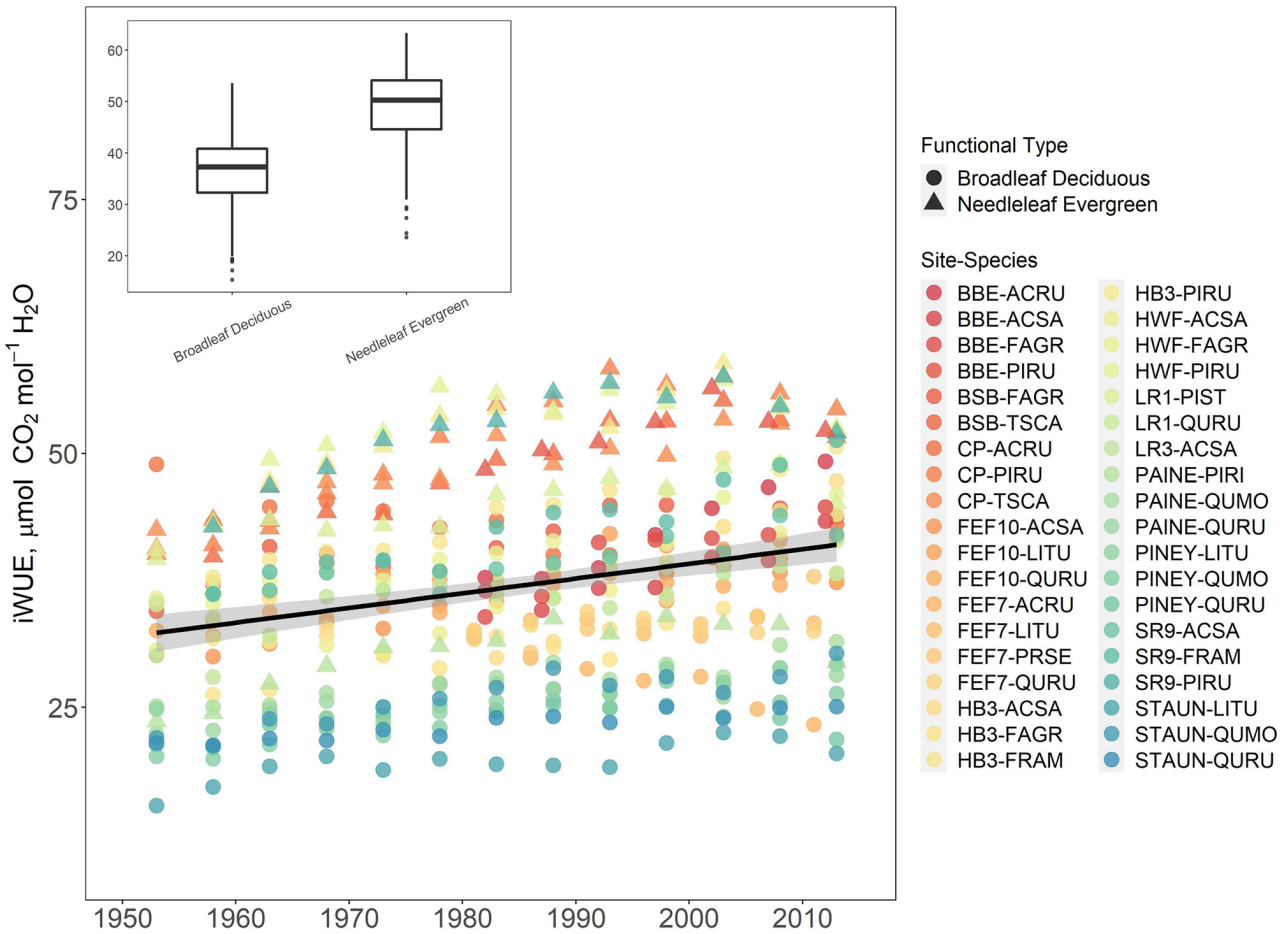
iWUE chronologies for all site-species combinations between 1950 and 2014. The fitted line represents the linear trend in iWUE over this time period (slope = 0.14 ± 0.5, *R*^2^ = 0.07, *p* < 0.0001). Triangles represent values for needleleaf evergreen species while circles represent values for broadleaf deciduous. Boxplots in the inset represent distributions of iWUE across all years for each leaf type, where boxes show the interquartile range. A full list of site and species codes can be found in the supplemental materials

When all site-species combinations were considered between 1951 and 2011 (the period of maximum overlap for all site-species combinations), iWUE increased 22.3% (Fig. 2), coinciding with a 25.2% increase in *C*_*a*_ over the same time period. Mean iWUE of needleleaf evergreen species was higher than that of broadleaf deciduous species over the entire study period (Fig. 2).

iWUE increased during the study period for both broadleaf deciduous and needleleaf evergreen species, but the temporal patterns differed markedly. Modeled fit for evergreen iWUE significantly better was significantly better with a GAM than a linear model (*R*^2^ = 0.82, AIC = 124 for the GAM, *R*^2^ = 0.65, AIC = 190 for the linear model; Table 2). The broadleaf deciduous iWUE chronologies exhibited linear behavior and a GAM therefore did not produce a better model fit (*R*^2^ = 0.49, AIC = 687 for the GAM, *R*^2^ = 0.49, AIC = 687 the linear model). Piecewise regression revealed a breakpoint in needleleaf evergreen iWUE in 2002 (*p* < 0.0001, 95% CI [1999, 2004]), after which iWUE declined (pre-breakpoint standardized slope = 0.06 ± 0.007, *R*^2^ = 0.78, *p* < 0.0001; post-breakpoint standardized slope = −0.09 ± 0.03, *R*^2^ = 0.57, *p* < 0.0001; Figure S2). In contrast, broadleaf deciduous iWUE increased linearly throughout the study period with no detectable breakpoint (standardized slope = 0.037 ± 0.004, *p* < 0.0001; Figure S2), suggesting that the linear model is more appropriate for broadleaf deciduous species.

**Table 2:** Summary statistics for linear and generalized additive models fit to iWUE chronologies of broadleaf deciduous and needleleaf evergreen species from 1953 to 2014

	Leaf functional type	Slope ± 95% CI	Adjusted *R*^2^	*p*-value	AIC
Linear model	Broadleaf Deciduous	0.037 ± 0.004	0.49	< 0.0001	687
	Needleleaf Evergreen	0.04 ± 0.006	0.65	< 0.0001	190
GAM	Broadleaf Deciduous	NA	0.49	< 0.0001	687
	Needleleaf Evergreen	NA	0.82	< 0.0001	124

**Fig. 3:**
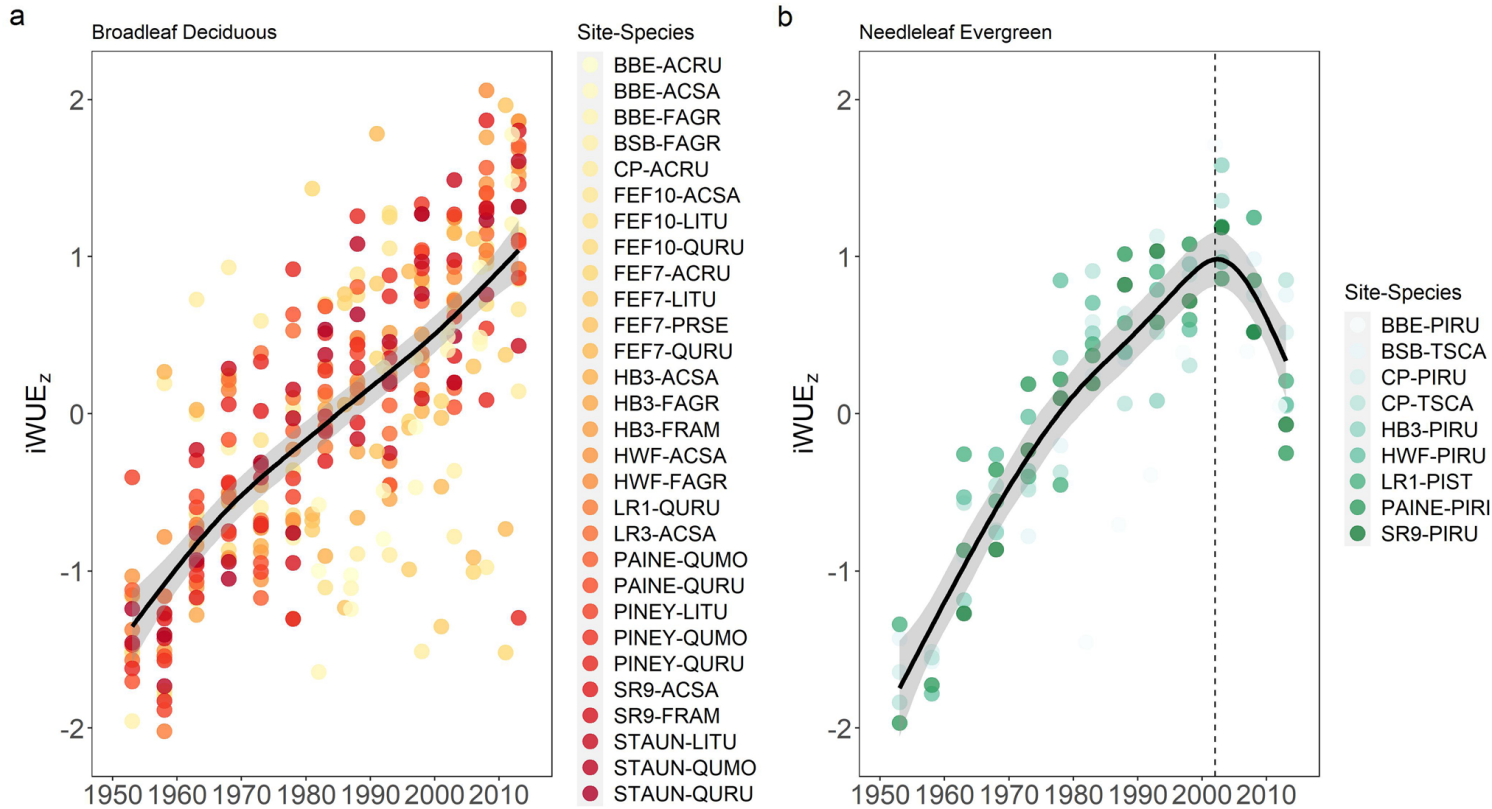
Temporal trends in standardized iWUE chronologies (z-scores) modeled with cubic regression GAMs for evergreen needleleaf species (**A**) and broadleaf deciduous species (**B**). Chronologies were either originally analyzed in 5-year increments or analyzed annually and aggregated into five-year bins prior to model fitting. The black line represents the fitted values for each leaf functional type category, while shaded areas represent 95% confidence intervals. Piecewise linear regression analysis was subsequently conducted on the chronologies for each leaf type, where a statistically significant breakpoint was identified in 2002 (± 3) for evergreen needleleaf species. There was no detectable breakpoint in broadleaf deciduous species. The dashed line represents the breakpoint in iWUE for each leaf type for evergreen needleleaf species

**Fig. 4:**
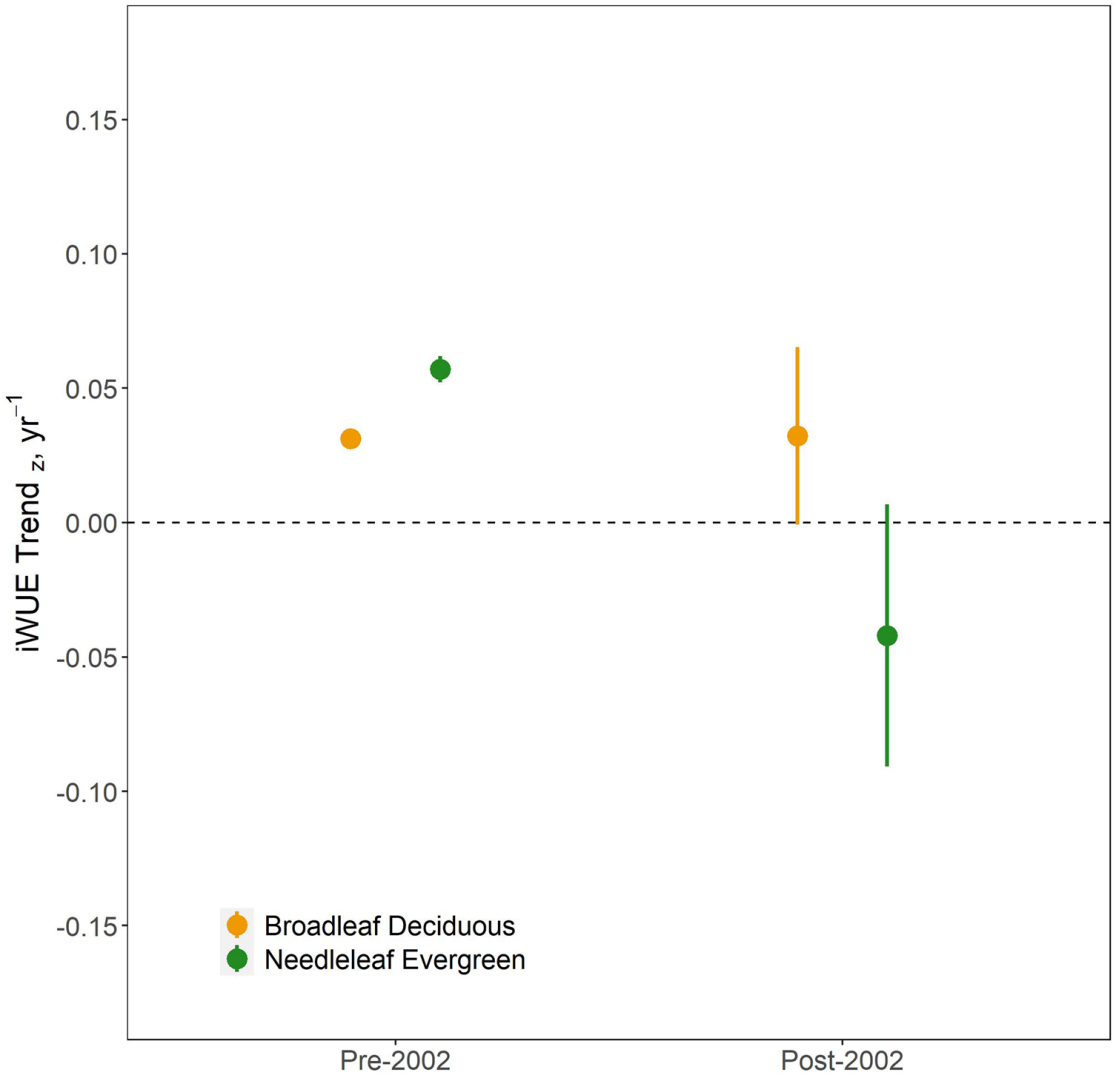
Standardized linear trends for trees of different leaf types prior to, and after, the 2002 iWUE breakpoint for evergreen species. Dots represent the mean trend while error bars represent 95% confidence intervals

Notably, evergreen iWUE increased more than twice as fast as broadleaf deciduous iWUE prior to 2002 (*p* < 0.0001), but that pattern reversed in more recent decades—after 2002, there was a negative trend in evergreen iWUE, while the rate of iWUE increase in broadleaf deciduous species remained unchanged (Figs. 3 and 4). When considering only broadleaf deciduous trees over the entire study period, there was no difference in iWUE trends between diffuse porous species, which tend to exhibit isohydric stomatal behavior, and ring porous species, which tend to exhibit anisohydric stomatal behavior (Figure S2).

### iWUE trends and environmental drivers

There was substantial intraspecific variation in iWUE trends among sites between 1980 and 2014, with some species exhibiting either significant positive or negative iWUE trends at different locations (Table S3). For example, red maple (*Acer rubrum*) iWUE increased significantly at BBE in Maine (slope 0.45 µmol mol^−1^ yr^−1^, *p* < 0.01), but decreased significantly in FEF7 in West Virginia (slope −0.28 µmol mol^−1^ yr^−1^, *p* < 0.01).

Across sites, deposition of N, S, ozone, and N + S critical loads exceedance covaried spatially, with generally higher levels of atmospheric pollution at sites in the mid-Atlantic than in the northeastern United States (Figure S3). This co-occurrence is consistent with the fact that oxidized N and S deposition often arise from the same pollution sources (Sullivan et al. 2018), and oxidized N is a key atmospheric precursor of ozone (Placet et al. 2000). We also found significant negative correlations between the slope of iWUE trends for all site-species combinations and atmospheric ozone (*r* = −0.42, *p* < 0.05), total N and S deposition (*r* = −0.46, *p* < 0.05), and exceedance of N and S critical loads for soil acidification (*r* = −0.34, *p* < 0.05; Figure S3).

### Environmental controls on iWUE trends

**Fig. 5:**
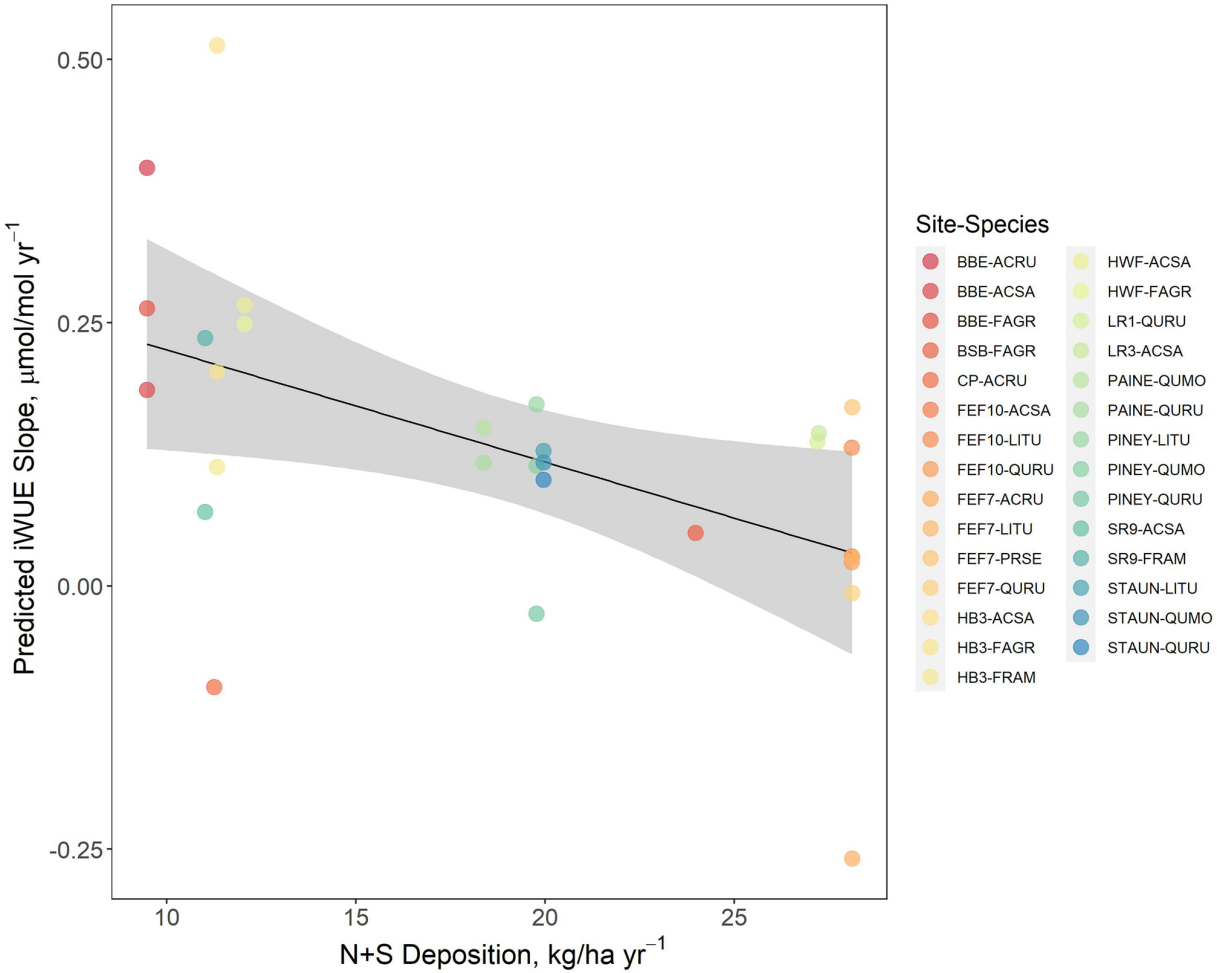
Partial residuals of air pollution mixed effects model depicting the predicted effects of total N + S deposition for all broadleaf deciduous site-species combinations. Points represent partial residual estimates of iWUE slope, and the shaded ribbon represents the 95% confidence interval

The comparison of models including climate, air pollution, and climate + air pollution explained 38, 48, and 51 percent of variance in iWUE slopes across all broadleaf deciduous site-species combinations, respectively (Table 2). The most parsimonious model included only total N + S deposition and ozone, with N + S deposition having a significant negative effect on the magnitude of iWUE trends, even after controlling for effects of ozone and species-specific iWUE responses (Table 2, Fig. 5). While Pearson’s correlation analyses indicated a strong positive correlation between N + S deposition and ozone (Figure S2), VIF was 1.4 for both variables, suggesting multicollinearity did not significantly affect estimation of model coefficients.

## Discussion

When considering all site-species combinations, our estimates of iWUE increased 22.3% between 1951 and 2011, coincident with a 25.2% increase in C_a_ (Fig. 2). This suggests that, on the whole, iWUE of temperate forest species in the eastern US has increased proportionally with C_a_, consistent with trees maintaining a constant *C*_*i*_/*C*_*a*_ ratio (Voekler et al. 2016). However, we observed substantial variability in iWUE trends between plant functional types and within species among sites, which indicates that both tree functional characteristics and environmental variables have modulated how trees respond to increasing *C*_*a*_.

### Differences in iWUE trends by tree functional characteristics

Broadleaf deciduous and needleleaf evergreen species exhibited divergent temporal patterns in iWUE. Prior to 1991, iWUE of needleleaf evergreen species increased more rapidly than that of broadleaf deciduous species (Figs. 2 and 3). After 1991 this pattern reversed—iWUE of broadleaf deciduous species continued to increase, while evergreen iWUE plateaued. While some models predict that iWUE should increase proportionally with *C*_*a*_, failure to account for the nonlinear behavior in evergreen iWUE response to *C*_*a*_ would result in a 32% overestimation of iWUE for this functional group for the time period 1991–2014. A pattern of saturating conifer iWUE in northeastern North America has been attributed to more pluvial conditions since the 1970s (Rayback et al. 2020; Belmecheri et al. 2021), and also declines in atmospheric deposition of S over roughly the same time period, which may have stimulated *g*_*s*_ more than *A* via improved stomatal function (ThoMas et al. 2013; Mathias and Thomas 2018; Rayback et al. 2020). Our observation of divergent iWUE trajectories of needleleaf evergreen and broadleaf deciduous species suggests distinct responses to recent environmental changes between species of the two leaf functional types that dominate eastern North American forests.

iWUE of needleleaf evergreen species increased faster than broadleaf deciduous species prior to 1991, consistent with evidence that gymnosperm species are more sensitive to increases in CO_2_ (Niinemets et al. 2011). However, the divergent iWUE trends, we observed between species of different leaf functional types (Fig. 3) contrast with recent studies that have shown that iWUE of evergreen species has increased more rapidly than deciduous species across multiple biomes (Soh et al. 2019), including temperate forests (Gharun et al. 2021), over recent decades. Adams et al. (2020) reported that rates of iWUE increase relative to continuing increases in C_a_ are diminishing globally, but that iWUE increased more rapidly in gymnosperms than angiosperms between 1966 and 2000. It remains to be seen whether the nonlinear iWUE pattern observed in conifers is a result of the particular combination of environmental changes experienced by trees in the eastern United States in recent decades, or whether these species have reached some maximum water use efficiency, either due to saturation of* A*, or inability to lower g_s_ (Voekler et al. 2016). The experimental evidence suggests that angiosperms may have evolved mechanisms to lower their *g*_*s*_ when *C*_*a*_ exceeds ~ 380 ppm that are absent in gymnosperms (Brodribb et al. 2009). *C*_*a*_ surpassed 380 ppm in 2006 (Belmecheri and Lavergne 2020), roughly coinciding with observed decline in needleleaf evergreen iWUE that began circa 2002. Thus, diminishing sensitivity *C*_*a*_ may partially explain the decline in iWUE observed in conifer species after 2002. Continued monitoring of iWUE across species with a diverse range of functional traits is needed to accurately project vegetation-climate feedbacks as *C*_*a*_ continues to increase.

### Environmental controls on iWUE trends

Across sites and species, both climate and atmospheric pollution variables are related to the magnitude of iWUE trends (Figure S3). However, mixed effects models revealed that differences in atmospheric pollution among sites explain substantially more variance in iWUE trends than differences in climate (Table 2). While both precipitation and VPD are important controls on the interannual variability in iWUE (Andreu-Hayles et al. 2011; Levesque et al. 2017), and prolonged drought or pluvial conditions can also modulate its long-term trajectory (Belmecheri et al. 2021; Kannenberg et al. 2021), we found that across climate and atmospheric pollution gradients, atmospheric pollution (which ranged from 4.7 to 11.9 kg N ha^−1^ yr^−1^ and 4.8 to 18.6 kg S kg ha^−1^ yr^−1^) exerted stronger influence on iWUE trends. Higher N and S deposition loads were associated with smaller increases (or in some cases decreases) in iWUE across all broadleaf deciduous site-species combinations, and also within some individual species. For example, we observed negative iWUE trends since 1980 in red maple (*Acer rubrum*) at the Fernow Experimental Forest in West Virginia (watershed name: FEF7), the highest deposition site, and positive iWUE trends in this species at sites in New England that received lower N and S deposition loads (Table S3).

N is often a growth-limiting nutrient in temperate forests (Groffman et al. 2018; ThoMas et al. 2010; Vadeboncoeur 2010), and N deposition may enhance iWUE by enabling increased *A*_*max*_ (Jennings et al. 2016; Adams et al. 2021; Gharun et al. 2021), despite the fact that elevated N deposition contributes to soil acidification (Gilliam et al. 2018). The low levels of S deposition may promote growth of some conifer species (Fenn et al. 2020), but S is primarily an acidifying agent in temperate forests of eastern North America, where it is associated with reduced tree growth in many species (Horn et al. 2018; Malcomb et al. 2020). Negative effects of N and S deposition on iWUE trends were observed even after controlling for the effects of species and ozone (Table 3, Fig. 5). This suggests that adverse impacts of acid deposition, such as foliar nutrient leaching (Sayre and Fahey (Sayre et al., 1999); Schaberg et al. 2001) and/or soil acidification effects such as base cation depletion and elevated phytotoxic aluminum (Likens et al. 1996; de Vries et al. 2003), outweighed N fertilization effects on iWUE. Disentangling direct and indirect impacts of N and S deposition on tree physiology remains a challenge, but it is notable that significant negative correlations were observed between both iWUE trends and total N and S deposition (*r* = −0.46, *p* < 0.05) and N and S critical loads exceedance for forest soil acidification (*r* = −0.34, *p* < 0.05; Figure S3). This implies that trees may be impacted by both leaf exposure to deposition and soil acidification, both of which can result in oxidative stresses that impair *A* (St. Clair et al. 2005; Wellburn 1990). These results suggest that trees subjected to the related, but mechanistically distinct, stressors of direct acid deposition and soil acidification may have diminished capacity to increase iWUE in response to increasing *C*_*a*_.

**Table 3:** Comparison of linear mixed effects models including climate, atmospheric pollution, and climate + pollution variables on the magnitude of iWUE trends of broadleaf deciduous species between 1980 and 2014

Parameter	Climate + Pollution	Climate	Pollution
Precipitation_JJA_	−0.04 ± 0.03	−0.09 ± 0.03*	–
VPD_JJA_	−0.01 ± 0.04	−0.07 ± 0.03*	–
Total N + S Deposition	−0.05 ± 0.06	–	−0.08 ± 0.03*
Ozone_JJA_	−0.04 ± 0.05	–	−0.05 ± 0.03
AICc	−6.38	2.8	−11.7
Marginal R^2^	0.50	0.38	0.48
Conditional R^2^	0.51	0.38	0.48

Model parameters were standardized prior to analyses and thus effects can be compared directly. In all models, tree species was specified as a random effect. Estimates for each variable ± the standard error are included, and significance is indicated by ^*^ *p* < 0.05. Marginal *R*^2^ values describe variance explained by fixed effects, while conditional *R*^2^ values describe variance explained by fixed and random effects

Ozone also has phytotoxic effects that can impair stomatal function resulting in reduced *A* (Wittig et al. 2009) and enhanced *g*_*s*_ (McLaughlin et al. 2007), although tree species vary in their sensitivity to ozone exposure. Similar to N and S deposition, there was a relatively strong negative correlation (*r* = −0.42) between ozone concentrations and iWUE trends across sites, and the effect of ozone on iWUE trends was smaller than the effects of N and S deposition (Table 2). While N and S deposition appear to be a stronger control on iWUE trends than ozone in this study, the magnitude and direction of effects were similar enough that we cannot rule out the possibility that ozone may have dampened the rate of iWUE increase at high exposure sites in recent decades, or that ozone may have equal or greater importance in other settings (e.g., sites with less acid deposition and greater ozone).

This analysis of trends in, and drivers of, tree iWUE in temperate forests of the eastern United States revealed distinct iWUE patterns between needleleaf evergreen and broadleaf deciduous trees species in the late 20th and early twenty-first centuries: after increasing at similar rates from the 1950s through the 1980s, iWUE of needleleaf evergreen species began to plateau around 1990, while broadleaf deciduous iWUE continued to increase. These results add to a growing body of evidence suggesting relatively weak *C*_*a*_ forcing of iWUE in needleleaf evergreen species in eastern North America in recent decades (Mathias and Thomas 2018; Rayback et al. 2020; Belmecherri et al. 2021). In light of observations of increased evapotranspiration in eastern US forests in recent decades (Vadeboncoeur et al. 2018; Hwang et al. 2020; Green et al. 2021), whether weakening iWUE trends in conifers have contributed to this phenomenon warrants continued study. Finally, across sites and species, we found that atmospheric deposition of N and S was negatively related to the magnitude of iWUE trends, suggesting that physiological stresses associated with acidic deposition may inhibit trees from responding optimally to increasing *C*_*a*_. Our results highlight the importance of tree functional traits and atmospheric pollution in regulating temperate forest iWUE, and consequently landscape-scale water, carbon, and energy budgets, with potentially significant implications for the carbon and water balance of ecosystems that account for two-thirds of terrestrial productivity in the United States each year (Lu et al. 2015).

## Supplementary Information

Below is the link to the electronic supplementary material.

Supplementary file1 (DOCX 335 KB)

## Data Availability

Data used in preparation of this manuscript can be accessed in a University of Virginia Dataverse repository at 10.18130/V3/HGCF8D.
