# Supplementary material for: Divergent water use efficiency trends among eastern North American temperate tree species
Source: Oecologia. 2025 Jul 29;207(8):137. doi: 10.1007/s00442-025-05753-w (PMC12307545; doi:10.1007/s00442-025-05753-w)
Supplement: Supplementary file 1 — Supplementary file1 (DOCX 335 KB) [file 442_2025_5753_MOESM1_ESM.docx]

***Electronic Supplementary Material***

**Article title:** Divergent water use efficiency trends among eastern North American temperate tree species

Authors: Jacob D. Malcomb, Howard E. Epstein, Matthew A. Vadeboncoeur, Daniel L. Druckenbrod, Matthew Lanning, Lixin Wang, Heidi Asbjornsen, Todd M. Scanlon

This supplement includes tables containing information about the source of ozone monitoring data (Table S1), detailed information about the trees core samples used to derive iWUE chronologies (Table S2), and Sen’s slopes and p-values from Mann-Kendal tests for trends in iWUE chronologies. Figures provide further information on differences in iWUE trends among trees with different functional traits (Figures S1 & S2) and results of correlation analyses assessing relationships between iWUE trends and environmental predictors (Figure S3).

**Table S1.** Site codes for ozone monitoring stations.

| **Tree Sampling Site** | **Ozone Monitoring Site Code** |
| --- | --- |
| Bear Brook East | 230194008 |
| Biscuit Brook | 361111005 |
| Cone Pond | 330099991 |
| Fernow WS7 | 540939991 |
| Fernow WS 10 | 540939991 |
| Hubbard Brook W3 | 330099991 |
| Huntington Forest | 360310003 |
| Leading Ridge 1 | 420279991 |
| Leading Ridge 3 | 420279991 |
| Paine Run | 511130003 |
| Piney River | 511130003 |
| Sleeper's River W9 | 500070007 |
| Staunton River | 511130003 |

**Table S2.** Details of tree species sampled at each site, number of trees represented in isotope chronologies, and time increment over which samples were processed.

Notes: Year increment refers to whether samples were processed in one of five-year increments. Composite sample indicates whether samples from different cores were combined prior to isotope analysis (Yes), or whether a mean iWUE chronology was calculated for the site-species combination after isotope analysis (No).

**Table S3.** Sen’s slopes and p-values from Mann-Kendall test for trend for all site-species combinations from ~1980-2014.

| **Site** | **Species** | **Leaf Type** | **Sen's Slope** | **P-value** |
| --- | --- | --- | --- | --- |
| BBE | ACRU | Broadleaf Deciduous | 0.456 | **0.007** |
| BBE | ACSA | Broadleaf Deciduous | 0.245 | **0.016** |
| BBE | FAGR | Broadleaf Deciduous | 0.322 | **0.003** |
| BBE | PIRU | Needleleaf Evergreen | 0.159 | 0.072 |
| BSB | FAGR | Broadleaf Deciduous | 0.058 | 0.764 |
| BSB | TSCA | Needleleaf Evergreen | 0.108 | 0.230 |
| CP | ACRU | Broadleaf Deciduous | -0.012 | 0.764 |
| CP | PIRU | Needleleaf Evergreen | -0.015 | 1.000 |
| CP | TSCA | Needleleaf Evergreen | 0.076 | 0.548 |
| FEF10 | ACSA | Broadleaf Deciduous | 0.011 | 1.000 |
| FEF10 | LITU | Broadleaf Deciduous | 0.114 | 0.133 |
| FEF10 | QURU | Broadleaf Deciduous | 0.005 | 0.764 |
| FEF7 | ACRU | Broadleaf Deciduous | -0.276 | **0.007** |
| FEF7 | LITU | Broadleaf Deciduous | 0.010 | 1.000 |
| FEF7 | PRSE | Broadleaf Deciduous | 0.153 | **0.007** |
| FEF7 | QURU | Broadleaf Deciduous | -0.024 | 0.368 |
| HB3 | ACSA | Broadleaf Deciduous | 0.598 | **0.003** |
| HB3 | FAGR | Broadleaf Deciduous | 0.288 | 0.072 |
| HB3 | FRAM | Broadleaf Deciduous | 0.198 | **0.016** |
| HB3 | PIRU | Needleleaf Evergreen | -0.007 | 1.000 |
| HWF | ACSA | Broadleaf Deciduous | 0.304 | **0.003** |
| HWF | FAGR | Broadleaf Deciduous | 0.286 | 0.072 |
| HWF | PIRU | Needleleaf Evergreen | -0.059 | 0.548 |
| LR1 | PIST | Needleleaf Evergreen | 0.082 | 0.368 |
| LR1 | QURU | Broadleaf Deciduous | 0.102 | **0.035** |
| LR3 | ACSA | Broadleaf Deciduous | 0.110 | 0.230 |
| PAINE | PIRI | Needleleaf Evergreen | -0.028 | 0.764 |
| PAINE | QUMO | Broadleaf Deciduous | 0.086 | 0.133 |
| PAINE | QURU | Broadleaf Deciduous | 0.054 | 0.368 |
| PINEY | LITU | Broadleaf Deciduous | 0.110 | **0.016** |
| PINEY | QUMO | Broadleaf Deciduous | 0.052 | 0.368 |
| PINEY | QURU | Broadleaf Deciduous | -0.089 | 0.072 |
| SR9 | ACSA | Broadleaf Deciduous | 0.118 | **0.016** |
| SR9 | FRAM | Broadleaf Deciduous | 0.284 | **0.016** |
| SR9 | PIRU | Needleleaf Evergreen | -0.045 | 0.764 |
| STAUN | LITU | Broadleaf Deciduous | 0.066 | 0.368 |
| STAUN | QUMO | Broadleaf Deciduous | 0.055 | 0.548 |
| STAUN | QURU | Broadleaf Deciduous | 0.039 | 0.230 |

Notes: All chronologies were converted to 5-year mean increments before calculation of Sen’s slope. Bold values indicated p<0.05.


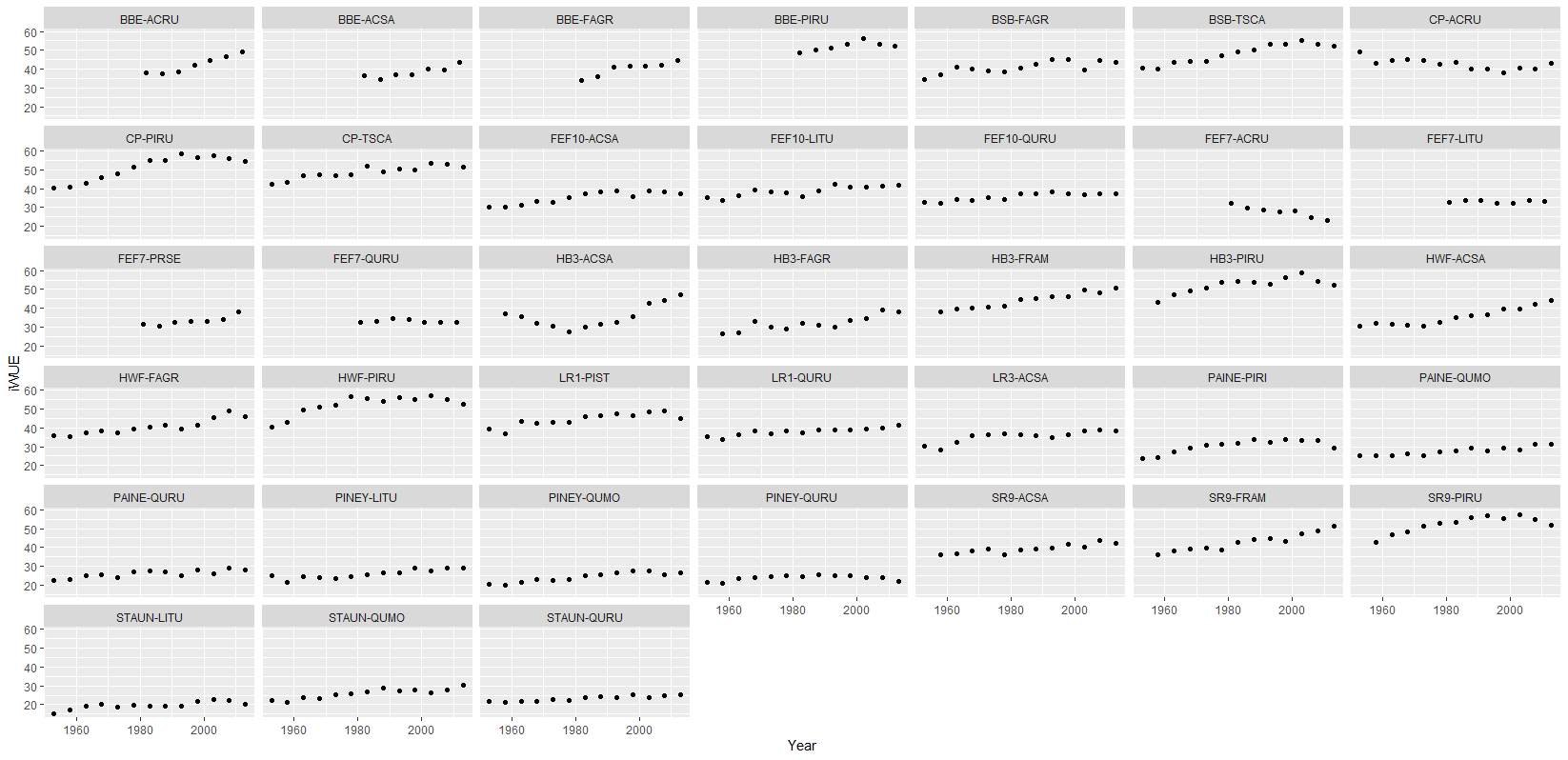


**Figure S1**. iWUE chronologies after binning annual chronologies into five-year increments for temporal consistency.


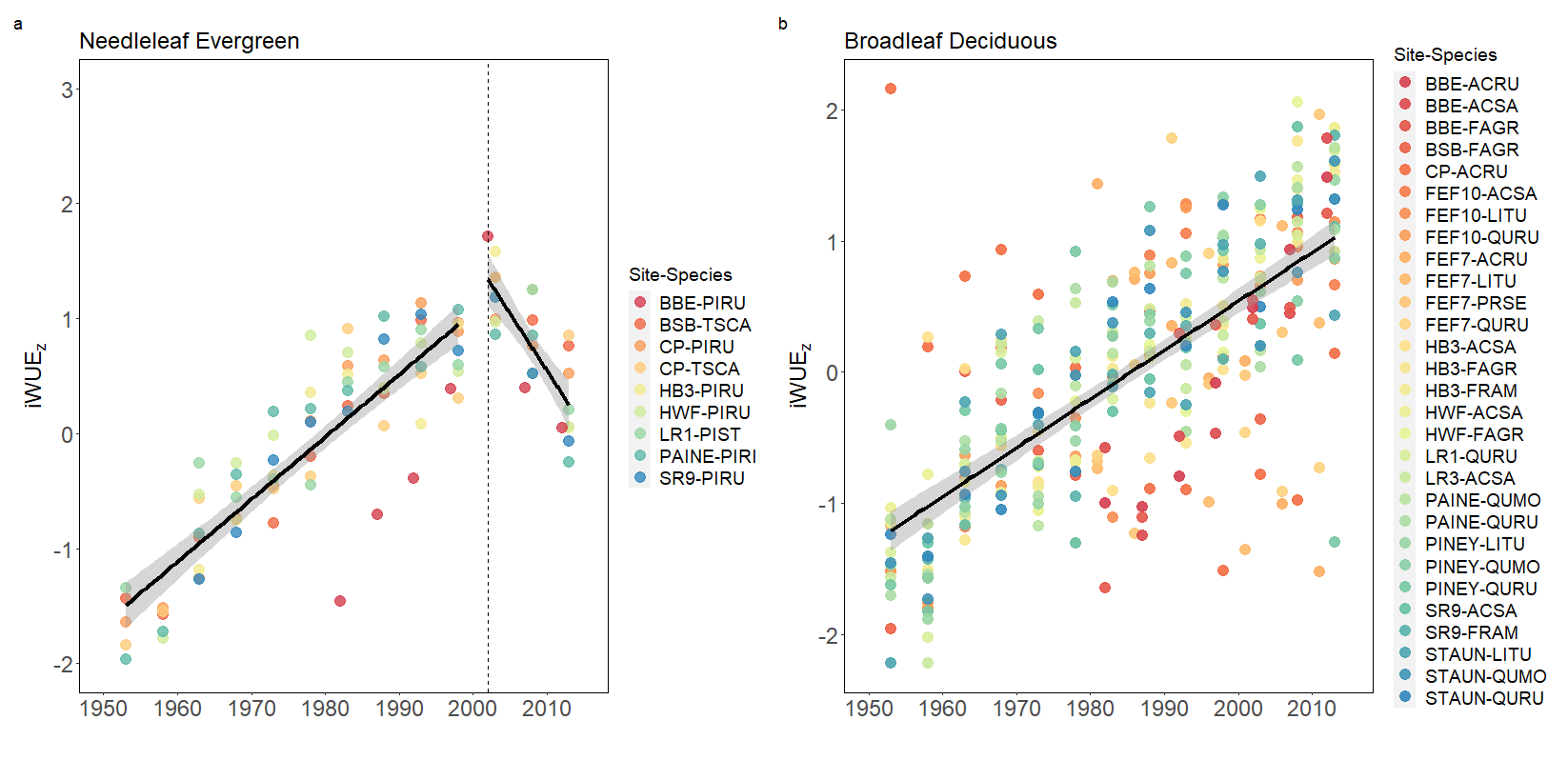


**Figure S2.** iWUE chronologies plotted using piecewise regression (Panel A), where a significant breakpoint was identified in 2002.


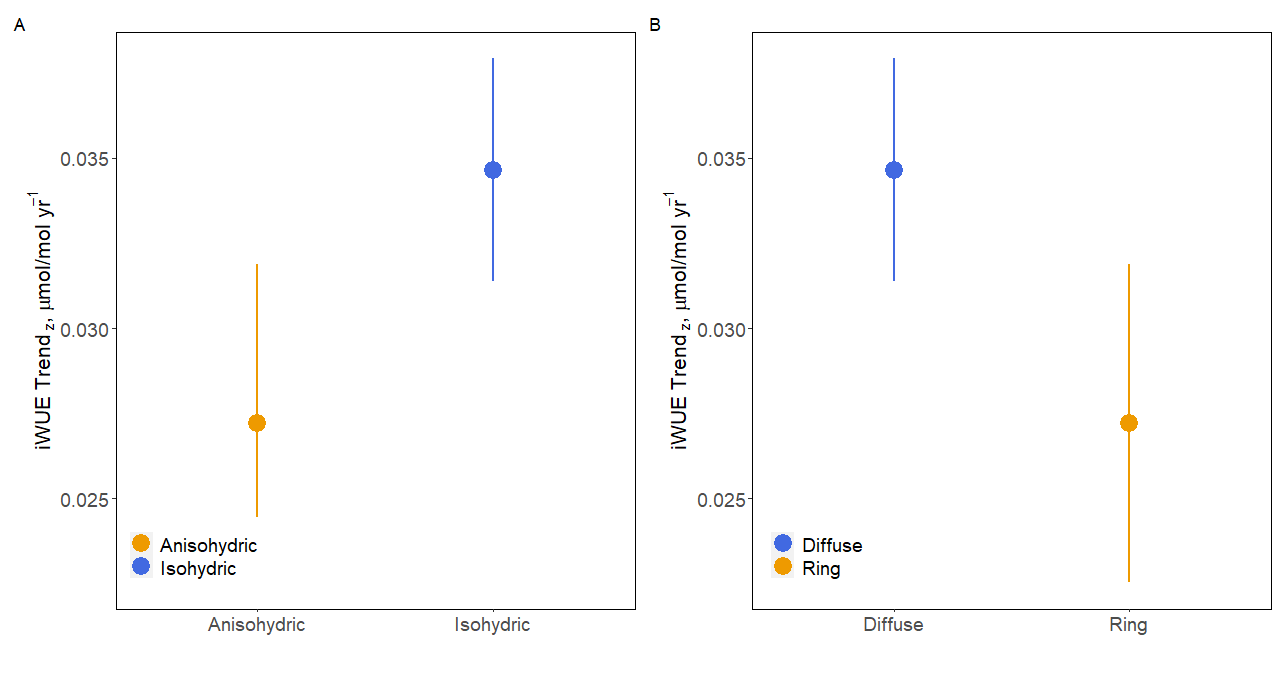


**Figure S3.** Comparison of iWUE trends over the entire study period (1950-2014) by stomatal behavior (A) and xylem anatomy (B) in broadleaf deciduous species. The difference between trends is not significant for either functional trait.


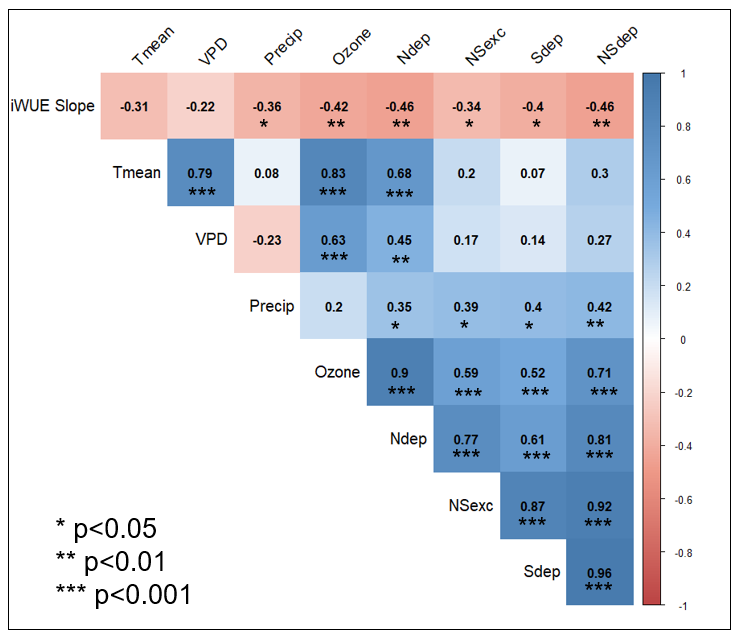


**Figure S4.** Pearson’s correlation coefficient between iWUE slopes and potential environmental drivers across all site-species combinations. Tmean=mean June-August temperature, VPD=mean June-August VPD_max_, Precip=mean June-August precipitation, Ozone=mean June-August ozone, Ndep=mean annual nitrogen deposition, NSexc=exceedance of N+S critical loads for forest soil acidification, Sdep=mean annual sulfur deposition, and NSdep=mean annual total N+S deposition. Statistical significance is denoted by *, where *p<0.05, **p<0.01, and ***p<0.001.
